# Overweight Mice Show Coordinated Homeostatic and Hedonic Transcriptional Response across Brain

**DOI:** 10.1523/ENEURO.0287-18.2018

**Published:** 2019-01-08

**Authors:** I. De Toma, I. E. Grabowicz, M. Fructuoso, D. Trujillano, B. Wilczyński, M. Dierssen

**Affiliations:** 1 Cellular and Systems Neurobiology, Systems Biology Program, Centre for Genomic Regulation (CRG), The Barcelona Institute of Science and Technology, Barcelona 08003, Spain; 2 Universitat Pompeu Fabra (UPF), Barcelona 08002, Spain; 3 Institute of Informatics, Faculty of Mathematics, Informatics and Mechanics, University of Warsaw, 02-097 Warsaw, Poland; 4 Postgraduate School of Molecular Medicine, Medical University of Warsaw, 02-091 Warsaw, Poland; 5 Centro de Investigación Biomédica en Red de Enfermedades Raras (CIBERER), 46010 València, Spain

**Keywords:** chromatin, gene expression, hedonic and homeostatic responses, obesity, TADs

## Abstract

Obesogenic diets lead to overeating and obesity by inducing the expression of genes involved in hedonic and homeostatic responses in specific brain regions. However, how the effects on gene expression are coordinated in the brain so far remains largely unknown. In our study, we provided mice with access to energy-dense diet, which induced overeating and overweight, and we explored the transcriptome changes across the main regions involved in feeding and energy balance: hypothalamus, frontal cortex, and striatum. Interestingly, we detected two regulatory processes: a switch-like regulation with differentially expressed (DE) genes changing over 1.5-fold and “fine-tuned” subtler changes of genes whose levels correlated with body weight and behavioral changes. We found that genes in both categories were positioned within specific topologically associated domains (TADs), which were often differently regulated across different brain regions. These TADs were enriched in genes relevant for the physiological and behavioral observed changes. Our results suggest that chromatin structure coordinates diet-dependent transcriptional regulation.

## Significance Statement

Mice fed with free-choice access to chocolate mixture (CM) become overweight and compulsive, recapitulating what happens during obesity. For the first time, we correlated these physical and behavioual changes with the transcriptome in the frontal cortex and the striatum, involved in the hedonic “liking” associated to eating, and the hypothalamus, involved in the homeostasic regulation of food intake. We detected two groups of genes: some transcript were strongly deregulated in term of fold changes, while others were only subtly deregulated but were especially correlating with measurements associated with body weight and compulsivity. These genes were not randomly distributed but were positioned in chromatin domains, many of which rich in genes differentially coregulated across brain areas.

## Introduction

Overeating, leading to obesity, is a serious concern in developed countries. Obesity is a major public health threat leading to related diseases such as Type II diabetes or atherosclerosis, and increasing mortality (Di Angelantonio et al., 2016). The brain circuitry controlling eating in humans, and participating in obesity development, is modulated not only by homeostatic mechanisms regulating food intake and energy expenditure but also by reward, emotion/memory, attention, and cognitive systems (Saper et al., 2002). Those mechanisms are non-homeostatic with regard to the body’s metabolism and energetic balance and may lead to addictive-like behaviors, such as compulsive overeating and inflexibility on obesity development (Lee et al., 2012), and act as potent drivers of food seeking (Kenny, 2011). The hypothalamus controls the energy-driven component of feeding behavior, while other regions, such as the frontal cortex and the striatum, control reward-related aspects of food intake. These “metabolic” and “hedonic” brain areas need to be coordinated to allow a proper ingestive behavior and a balanced energy intake (Berthoud, 2012) and would be affected by facilitated access to energy-dense and palatable food (Berridge et al., 2010). This coordination among distant brain areas naturally uses multiple mechanisms, including cell-to-cell signaling and long-range projections among different brain regions (Atasoy et al., 2012; Sweeney and Yang, 2017). However, it also requires coordinated transcriptional regulation in various brain regions (Fenselau et al., 2017). Much of the transcriptional response associated to overeating remains to be studied, and the relational patterns in gene expression changes among different metabolic and hedonic-related brain regions are unknown.

One possibility would be that this regulation takes place in the context of topologically associated domains (TADs). TADs are chromosomal domains evolutionarily conserved across tissues and species. The genes present in TADs usually exhibit similar expression profiles (Dixon et al., 2012), forming coregulated clusters (Nora et al., 2012). Thus, we propose that the TAD structure orchestrates the gene expression changes across different brain regions, allowing both a coordinated and region-specific response across different brain regions.

Here, we explored the transcriptional profiles of frontal cortex, striatum, and hypothalamus, key brain areas involved in overeating, in mice fed with free choice of a high palatable and energy-dense diet, a model for overeating and unhealthy food consumption. We also measured physical and behavioral parameters to correlate them with transcriptional changes. Once we detected the genes changing their expression levels and correlating with body weight and behavior, we explored their distribution on TADs across brain regions.

## Materials and Methods

### Animals

We used sixteen C57BL/6 (Charles River) female mice, of five weeks of age at the beginning of the experiments. Mice were housed in individually ventilated cages (IVCs; Tecniplast) and PheCOMP cages (Multitake model, Panlab) in the Animal Facilities of the Barcelona Biomedical Research Park (PRBB, Barcelona, Spain) in controlled laboratory conditions with the temperature maintained at 22 ± 1°C and humidity at 55 ± 10% on a 12/12 h light/dark cycle (lights off 8 P.M.). Food and water were available *ad libitum*. Animal procedures were conducted in accordance with the local (law 32/2007) and European regulations (EU directive n° 86/609, EU decree 2001-486) and the Standards for Use of Laboratory Animals n° A5388-01 (NIH).

### Diet-induced weight gain

All mice were habituated to their cages for one week provided with food and water *ad libitum*. Then, they were allocated to the group receiving standard chow (SC) or chocolate mixture (CM), balanced by body weight and housed individually in special metabolic cages (see Feeding behavior analysis). During eight weeks, SC mice had access to SC mouse diet (Trans 23 diet, Mucedola) providing 10870 kJ/kg, and CM mice had a free choice access to SC and to a CM consisting of an equal weight of Mars, Bounty, Snickers, and Milka prepared as homogenous food pellets following a protocol previously described (Heyne et al., 2009). The chocolate provides 20595 kJ/kg with 52% of its energy from carbohydrate, 17% from protein, and 24% from fat. The experimental schedule is shown in Extended Data Figure 1-1.

10.1523/ENEURO.0287-18.2018.f1-1 Extended Data 1-1 Table with the behavioral and physical data collected. Download Extended Data 1-1, CSV file.

### Feeding behavior analysis

We used the PheCOMP multi-take metabolism cages (Panlab-Harvard Instruments) to obtain fine grain data for individualized mouse including the grams of food consumed, the number of meal events and the temporal distribution of the feeding bouts in a continuous recording (Espinosa-Carrasco et al., 2018). The system contains two foods dispensers. SC mice received standard rodent chow (SC) in both, whereas CM mice had one dispenser with SC and the other with CM. The location of each dispenser was counterbalanced between cages. From the quantitative data obtained by the PheCOMP cages, we calculated the energy intake, measured by multiplying the known energy content (kJ/g) of individual foods by the amount of food consumed. The eating rate (kJ/s) was obtained using the COMPULSE software (Panlab-Harvard Instruments).

### Test battery for the study of compulsivity

Tests were performed in a set order designed to minimize the effect of testing on following tests and with sufficient intertest intervals to provide an opportunity for the mouse to reestablish its previous feeding behavior and relieve any test-induced stress. The free-choice diet was suspended only during the “limited access to CM” and “CM adulteration” tests (5 d in total). Thereafter, the initial diet was reintroduced during 6 d, before the animals were killed.

### Temporally limited access to CM

We used limited access to CM to measure the binge-like behavior, as readout of compulsion induced by restricted access to the preferred food. SC and water were provided *ad libitum*. Access to CM was restricted to 1 h/d during the middle of the light phase for three consecutive days (Heyne et al., 2009). SC mice were also provided with CM during this hour. In CM mice, we compared the CM consumed during the period of access with the CM consumed in non-limited conditions. This value was obtained as the mean of 3 d of CM intake during the previous week of the battery of tests, at the same time (between 2 and 3 P.M.).

### CM adulteration

Chocolate adulteration provides information concerning flexibility of food intake under aversive conditions. Mice were given a free choice between SC and a pellet of the CM adulterated with quinine hydrochloride (Sigma-Aldrich) 1-g/kg food to give it a bitter taste. According to Heyne et al. (2009), flexible mice will avoid or decrease the intake of CM.

### Nestlet shredding test and grooming behavior

Mice were given a cotton square (Ancare) in their home cage under food-deprived conditions (water was still provided *ad libitum*) for 30 min during the middle of the light phase. The cotton was weighed before and after the test to provide a measure of nesting ability based on the amount of material the mouse had used to nestlet (Deacon, 2006). The grooming behavior was recorded (Biobserve), and the number and length of events were quantified by an investigator blind to the experimental condition.

### Statistical analysis of behavior

Repeated measures ANOVA was used for the comparison of the body weight evolution across the experimental weeks. Differences were considered significant at *p* < 0.05. All results are expressed in mean ± SEM. The statistical analysis was performed using the Statistical Package for Social Science program SPSS 12.0 (SPSS Inc).

### Gene expression

Frontal cortex, striatum, and hypothalamus, from SC and CM groups, were dissected on completion of an 11-d test battery and total RNAs extracted with QIAGEN’s RNeasy mini kit for hybridization with Agilent’s gene expression arrays (SurePrint G3 Mouse GE 8x60K array v1).

Cyanine-3 (Cy3)-labeled cRNA was prepared from 100 ng of total RNA using the LowInputQuick Amp Labeling kit Agilent 5190-2305 according to the manufacturer’s instructions, followed by RNAeasy column purification (QIAGEN). Dye incorporation and cRNA yield were checked with the NanoDrop ND-1000 Spectrophotometer.

After fragmentation, 600 ng of labeled cRNA from each sample was hybridized in *in situ* hybridization oven (Agilent) for 17 h at 65°C and washed during 1 min at room temperature in Gene Expression Wash buffer 1 (Agilent) and 1 min at 37°C with Gene Expression Wash buffer 2 (Agilent).

Scanned on an Agilent G2539A scanner at 3-μm resolution and 100% PMT. The intensity data of each individual hybridization were extracted and the quality was assessed with the Feature Extraction software 10.7 (Agilent). The intensity data of each individual hybridization were extracted, and the quality was assessed with the Feature Extraction software 10.7 (Agilent).

### Bioinformatic analysis

Intensity values were imported into R using the limma function read.maimages (Ritchie et al., 2015). Samples were background corrected and normalised using the normexp normalization: a convolution of normal and exponential distributions is fitted to the foreground intensities using the background intensities as a covariate, and the expected signal given the observed foreground becomes the corrected intensity (Shi et al., 2010). This results in a smooth monotonic transformation of the background subtracted intensities such that all the corrected intensities are positive. Background has been computed from the 95th percentile of the intensity of the negative control probes on each array, keeping probes that are at least 10% higher than the negative controls on at least four arrays (because there are four biological replicates). Values for within-array replicate probes are replaced with their average to have a “one value – one gene” matrix. We fitted a linear model by using both brain areas and diet as covariates, blocking for the mouse for taking into consideration the same provenance of the three brain regions. The values of the moderated *t* statistics were corrected for multiple-testing using the Benjamini–Hochberg correction (Benjamini and Hochberg, 1995).

Multidimensional scaling was performed using the limma plotMDS function. The distance between each pair of samples is the root mean square deviation for the top 500 genes (selected for each pair of samples). Distances on the plot can be interpreted as leading log2 fold change (log2FC), meaning the typical (root mean square) log2FC between the samples for the genes that distinguish those samples (Ritchie et al., 2015).

We used the matplotlib_venn python module and gplots R package (Warnes et al., 2016) for drawing the overlaps, and we assessed the significance of the overlaps with the Fisher’s exact test.

Probes were converted to entrez identifier by using the biomaRt package (Durinck et al., 2009) and gene ontology and pathway analysis were performed with the clusterProfiler package (Yu et al., 2012).

### Correlation with behavioral data

We correlated each of the gene expression microarray intensities with body weight and the behavioral data measured in our mice. Since we were interested both in the final body weight and in its increase, we performed a principal component analysis (PCA) with these two variables and extracted for each mouse the resulting values of principal component 1 to combine those variables in a unique measurement (Extended Data Fig. 3-1*C*). Similarly, we combined through PCA six other variables to have a unique measurement of compulsivity: grooming; nesting; the energy rate from days 1, 2, 3; and from the quinine adulteration test (Extended Data Fig. 3-1*B–F*). The variables within these two sets were correlated with the gene expression values. After this analysis, we finally selected five parameters to correlate: two set of PC1 values, body weight and compulsivity, and three behavioral measurements inflexibility, energy intake, and eating rate. We selected for further analyses only the correlating microarray probes changing between SC and CM mice at least by 10% and whose adjusted *p* value on *z* Fisher correction was lower than 0.05.

### Testing whether the gene expression fold changes fitted the TAD segmentation profile

We mapped genes with the Entrez identifiers using BiomaRt (Ensembl archive May, 2017) to the TAD borders as defined in Dixon et al. (2012) for cerebral cortex. TADs borders were defined using a Directionality Index method (Dixon et al., 2012). To test whether there was agreement between the differential expression profile in the three studied brain regions and the TAD segmentation, we performed three types of *in silico* permutation testing (Extended Data Fig. 4-1*A*). For the tests, we selected only the genes that were clustered within TADs containing five or more genes. In the first approach, genes were reassigned to random TADs; however, maintaining original gene numbers in particular TADs and using only the genes, which were within TADs in the original case. Second type of permutation involved changing borders of TADs by permuting the collection of pairs of TAD’s length + distance to the next TAD downstream, maintaining the original gene localizations on chromosomes. Each type of permutations was made 1000 times, and each time, Kruskal–Wallis test was performed. Finally, the *H* statistics from original data were compared with the averaged values from the permuted *H* statistics as well as compared with the decreasing rank of the permuted *H* values.

### Selecting regulated TADs

We defined as regulated TADs the TADs with a significantly higher number of DE or correlating genes across the three brain areas. Once mapped these genes to TADs, we used the PowerLaw R Package to check what kind of heavy-tail distribution the number of regulated genes per TAD approximated. We compared Poisson, Power-law, exponential, and log normal, finally selecting the log normal to select the TADs whose probability of finding by chance another TAD with a higher number of regulated genes was lower than 0.05.

### Testing whether responsive genes are coregulated within regulated TADs

To test whether the DE and correlating genes contained in regulated TADs clustered according to their fold change (e.g., upregulated genes in certain TADs, downregulated genes in other TADs), we performed a permutation test. We considered as responsive genes each gene DE or correlating. Our rationale was that in case of an equal number of upregulated and downregulated genes, if genes were randomly distributed along TADs, the difference between the number of upregulated and the number of downregulated genes had to be on average 0, while if the contrary were true, we would expect both TADs with a positive difference (more upregulated genes) and with a negative difference (more downregulated genes). Therefore, first we computed the absolute value of the differences between the number of upregulated and downregulated responsive genes for each TAD, and then we calculated the average observed deviation per regulated TAD, in each brain region. We then randomly shuffled 1000 times the responsive genes maintaining the number of genes per TAD fixed and recalculated the average deviations in each region. The *p* value was given by summing how many times we observed by chance (in the permuted datasets) a higher deviation than what observed in our data +1, divided by the number of permutation +1. Using only responsive genes for reassigning genes to regulated TADs, we assured that our results were significantly different from what expected by chance for a given pattern of fold changes. For instance, if upregulated genes among responsive were naturally more numerous, we would expect higher deviation from zero even if these genes were randomly distributed across regulated TADs, and therefore we took into account this higher probability of reassigning an upregulated gene in our permutations.

All the code for the bioinformatic analysis is reported as supplementary code used in this article 1-1. Analyses were performed with R version 3.5.0 (2018-04-23). Platform: x86_64-apple-darwin15.6.0 (64-bit). Running under: macOS High Sierra 10.13.4.

10.1523/ENEURO.0287-18.2018.f1 Extended Data 1 Zip file containing the R mark Down file with the code to reproduce all the analyses performed in R. Download Extended Data 1, ZIP file.

### Contact map

Contact-map was created using visualization tool DiffTAD (Zaborowski and Wilczynski, 2016), and the chromatin contacts data (TAD borders) come from the Hi-C experiment performed by Dixon et al. (2012).

### Data availability

Data have been uploaded on GEO with the reference GSE100012. The code to reproduce the analysis is available on Bitucket at https://bitbucket.org/ilario_de_toma/freechoice.

## Results

### Free access to chocolate induces overweight and compulsive overeating

**Figure 1.**
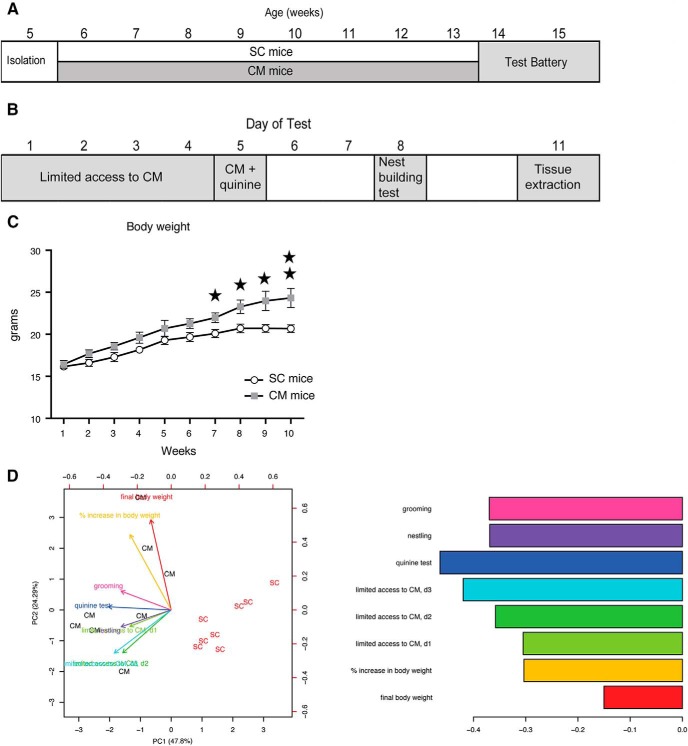
Free-access to a CM leads to body weight gain. ***A***, Experimental schedule showing the age of mice along the experiment. Note that during the test battery animals continued receiving the same diet as during the weight gain phase (6–15 weeks of age). ***B***, Detail of the standardized testing battery used and the days of administration of each test. ***C***, Body weight (in g) changes with time in SC (white circles) and CM (gray squares) mice along the 10 weeks of the experiment. ***D***, Biplot of PCA on SC and CM mice using bodyweight and eating-related behavioral variables indicated by colored arrows (left panel). Barplot showing the contribution of the variables to principal component 1 (right panel). Obesity was defined by the variables final body weight and percentage of body weight gain; compulsivity was evaluated by the CM intake during the 3 d of limited CM access, nest building behavior, and grooming; inflexibility was explained by the amount of CM consumed in the quinine test. d1: day1; d2: day2; d3: day3.

To investigate the effect of our experimental design on the brain transcriptome, we performed *in vivo* experiments and took measuraments from eight mice given free access to CM and SC (CM mice; Fig. 1*A*,*B*) and eight mice receiving SC (SC mice). CM mice increased their body weight on CM access (repeated measures ANOVA, *F*_(1,14)_ = 19.30; *p* = 0.001), whereas SC mice did not significantly change their weight along the experiment (Fig. 1*C*). There is a slight increase of body weight in both experimental groups during the first weeks, possibly reflecting the normal growth curve, but after eight weeks of free CM access, body weight was significantly higher in the CM group only. We also measured behavioral parameters to correlate them with transcriptional changes. The test battery included limited access to the chocolate, quinine test, nest building test (Fig. 1*B*). Moreover, we monitored in both groups the energy intake (kJ/kg), eating rate (mg/s), food intake during limited access and quinine test (g/kg of body weight/h), and grams of cotton in the nest building test, and the grooming time (s). We then checked whether these measurements were able to separate SC from CM mice using PCA (Fig. 1*D*). Interestingly, behavioral variables contributed to the separation of SC and CM mice along PC1, as much or even more than body weight related variables, suggesting that body weight changes in CM mice are accompanied by strong behavioral changes. Raw behavioral data are accessible in supplementary code used in this article 1-1.

### Transcriptional responses can be clustered by brain region and diet

**Figure 2.**
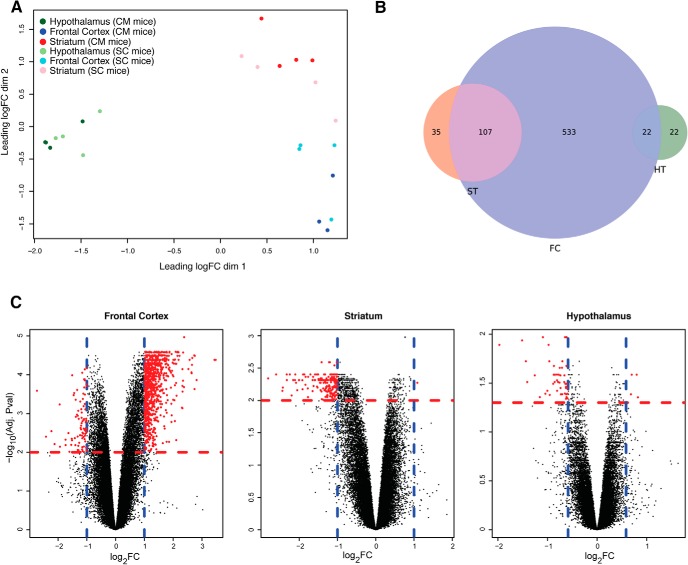
Differential expression analysis reveals that different brain areas present different transcriptional profiles when comparing SC and CM mice. ***A***, Multidimensional scaling plot with the top 500 most variable intergroup probes. HT indicates the hypothalamic region (green dots), ST the striatum region (red dots), FC the frontal cortex (blue dots), SC mice are represented with light colors, CM mice are colored in dark colors. ***B***, Venn diagram showing the overlap among DE genes used for the enrichment analysis in the three brain areas (absolute fold change ≥1.5, adjusted *p* < 0.05 for hypothalamus, absolute fold change ≥2 and adjusted *p* < 0.01 for the frontal cortex and the striatum). Colors represent the same brain areas as in ***A***. Circles’ areas are proportional to the gene counts. ***C***, Volcano plots for the three brain areas, on the *x*-axis log2 (fold changes), on the *y*-axis the significance (-log_10_ of the adjusted *p* value). Blue lines mark fold changes thresholds, red lines significance threshold. Each dot corresponds to a probe. Significant probes are marked in red.

We performed a microarray experiment to assess the effect of our experimental design on the transcriptional profile of three brain areas: the frontal cortex, the striatum, and the hypothalamus (four animals per group). Multidimensional scaling showed that the first leading dimension is mainly separating the hypothalamus from the frontal cortex and the striatum, indicating that the hypothalamic transcriptional profile diverges significantly from that of the striatum and frontal cortex, while the second dimension is further separating the frontal cortex from the striatum and, less perfectly, SC from CM mice (Fig. 2*A*).

When assessing the CM-SC contrast with a linear model, we found 662 differentially expressed (DE) genes on CM diet in the frontal cortex, 142 in the striatum, and 44 in the hypothalamus on setting specific threshold of fold changes and adjusted *p* value (Fig. 2*B*,*C*). Two thirds of the striatal and half of the hypothalamic DE genes significantly overlapped with frontal cortex DE genes. Instead, we found no overlap between the striatum, part of the reward system, and the hypothalamus, involved in homeostatic energy intake. Volcano plots of the overall transcriptomic changes showed that frontal cortex genes presented the higher absolute fold changes, followed by the striatum, while hypothalamic genes showed modest fold changes, indicating that weight gain led to a wider and stronger response (in term of differential expression) in the frontal cortex (Fig. 2*C*). Summary tables for the differential expression analyses are reported as Extended Data 2-1, 2-2, 2-3.

10.1523/ENEURO.0287-18.2018.f2-1 Extended Data 2-1 Differential expression analysis for the frontal cortex. Download Extended Data 2-1, CSV file.

10.1523/ENEURO.0287-18.2018.f2-2 Extended Data 2-2 Differential expression analysis for the striatum. Download Extended Data 2-2, CSV file.

10.1523/ENEURO.0287-18.2018.f2-3 Extended Data 2-3 Differential expression analysis for the hypothalamus. Download Extended Data 2-3, CSV file.

**Figure 3.**
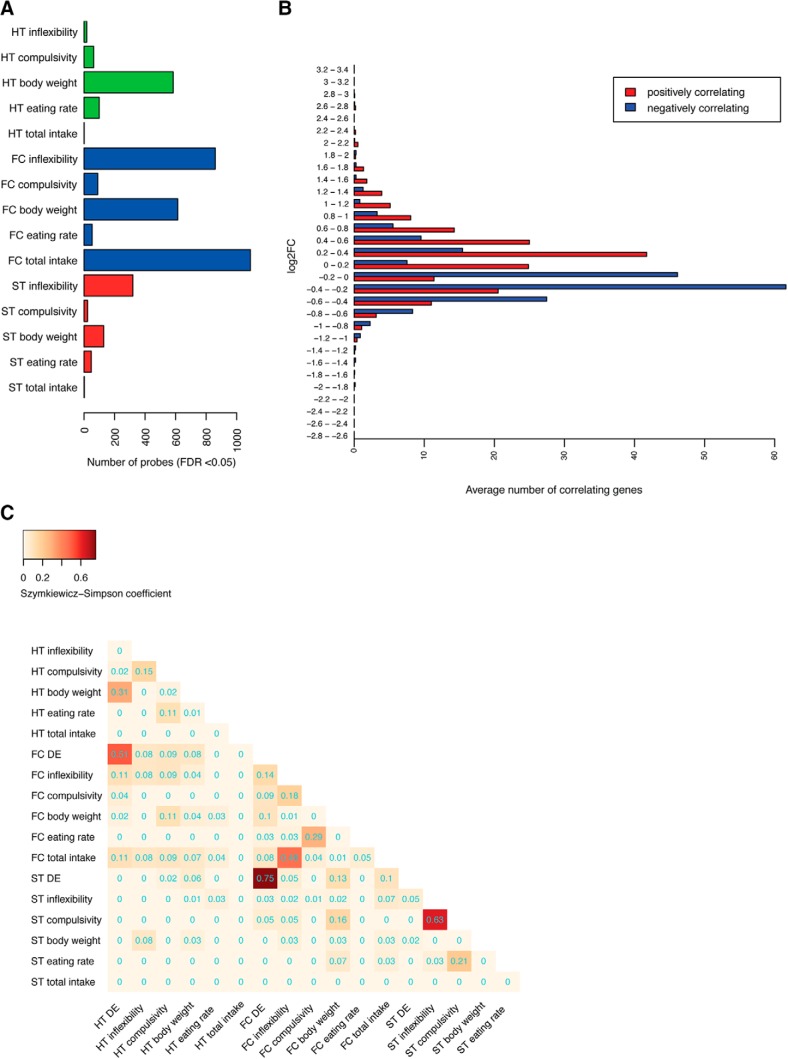
A subset of moderately expressed genes highly correlates with phenotypical changes. ***A***, Barplot showing the number of genes correlating for each of the phenotypic variables that were detected with a false discovery rate <5%. ***B***, Barplot showing the average number of genes with an absolute ρ higher than 0.9 for a given bin of log2FC. The averages were calculated across all brain areas and variables. The majority of correlating genes have log2FC within 0.2–0.4 ranges (positive correlation marked in red and negative in blue). ***C***, Heatmap showing the Szymkiewicz−Simpson overlap coefficient between DE genes and genes correlating with eating-related variables. Brain region acronyms are the same as in Figure 2. Color code according to the coefficient. HT indicates the hypothalamic region, ST the striatum region, FC the frontal cortex, and DE as differentially expressed. Extended information related to the correlation between transcriptional changes with the physical/behavioral alterations could be found in Extended Data Figures 3-1, 3-2, 3-3, 3-4, 3-5, 3-6, 3-7.

10.1523/ENEURO.0287-18.2018.f3-1 Extended Data Figure 3-1 Correlation of transcriptional with behavioral analysis. ***A***, Biplot of PCA on SC and CM mice using body weight variables indicated by colored arrows (top) showing the clear separation between CM and SC mice. Barplot showing the contribution of the body weight variables to the principal component 1 (bottom). ***B***, Biplot of PCA on SC and CM mice using behavioral variables indicated by colored arrows (top). Barplot showing the contribution of behavioral variables to the principal component 1 (bottom). ***C–E***, Individual values of the variables used for gene expression correlation in SC mice (red) and CM mice (black). Note that eating rate and total intake values were only available for three of the four individuals used for the transcriptome analysis. ***C***, Barplot showing the differences in CM energy intake of SC and CM mice in the quinine adulteration test. ***D***, Barplot showing the differences in total energy intake of SC and CM (CM mice). Data from one of the mice in the CM group were missing. ***E***, Barplot showing the differences in eating rate of SC (SC mice) and CM (CM mice). Download Figure 3-1, TIF file.

### Most of the genes highly correlating with behavioral variables show subtle expression changes

To determine which transcriptional changes were correlating with the physical/behavioral alterations, we tested the correlation of the overall gene expression changes (not only those DE) with the five parameters that mainly contributed to the observed intersample variance (see Materials and Methods). These parameters included both composite measurements: body weight (Extended Data Fig. 3-1*A*) and compulsivity (Extended Data Fig. 3-1*B*), and direct measurements: inflexibility (Extended Data Fig. 3-1*C*), total food intake, and eating rate (Extended Data Fig. 3-1*D*,*E*).

In each brain region, we identified sets of genes significantly correlating with specific behavioral/physical variables (Fig. 3*A*). The frontal cortex showed the highest number of genes correlating with total food intake, body weight, and inflexibility and, to a lesser extent, compulsivity and eating rate. In the hypothalamus, we detected a high number of genes correlating with body weight, while in the striatum, we found a lower number of correlating genes, mostly correlating with inflexibility.

10.1523/ENEURO.0287-18.2018.f3-2 Extended Data Figure 3-2 Genes correlating with inflexibility. ***A***, Dot plot showing the ρ score for inflexibility (*x*-axis) and the log2FC (*y*-axis) in frontal cortex. Each dot is a microarray probe. ***B***, Barplot showing how many genes with an absolute ρ higher than 0.9 for a given bin of log2FC. ***C***, Same as in ***B*** normalized for the total number of genes present in a given bin of log2FC. ***D–F***, Same as in ***A–C***for striatum. ***G–I***, Same as in ***A–C*** for hypothalamus Download Figure 3-2, TIF file.

10.1523/ENEURO.0287-18.2018.f3-3 Extended Data Figure 3-3 Genes correlating with compulsivity. ***A***, Dot plot showing the ρ score for compulsivity (*x*-axis) and the log2FC (*y*-axis) in frontal cortex. Each dot is a microarray probe. ***B***, Barplot showing how many genes with an absolute ρ higher than 0.9 for a given bin of log2FC. ***C***, Same as in ***B*** normalized for the total number of genes present in a given bin of log2FC. ***D–F***, Same as in ***A–C*** for striatum. ***G–I***, Same as in ***A–C*** for hypothalamus Download Figure 3-3, TIF file.

**Figure 4.**
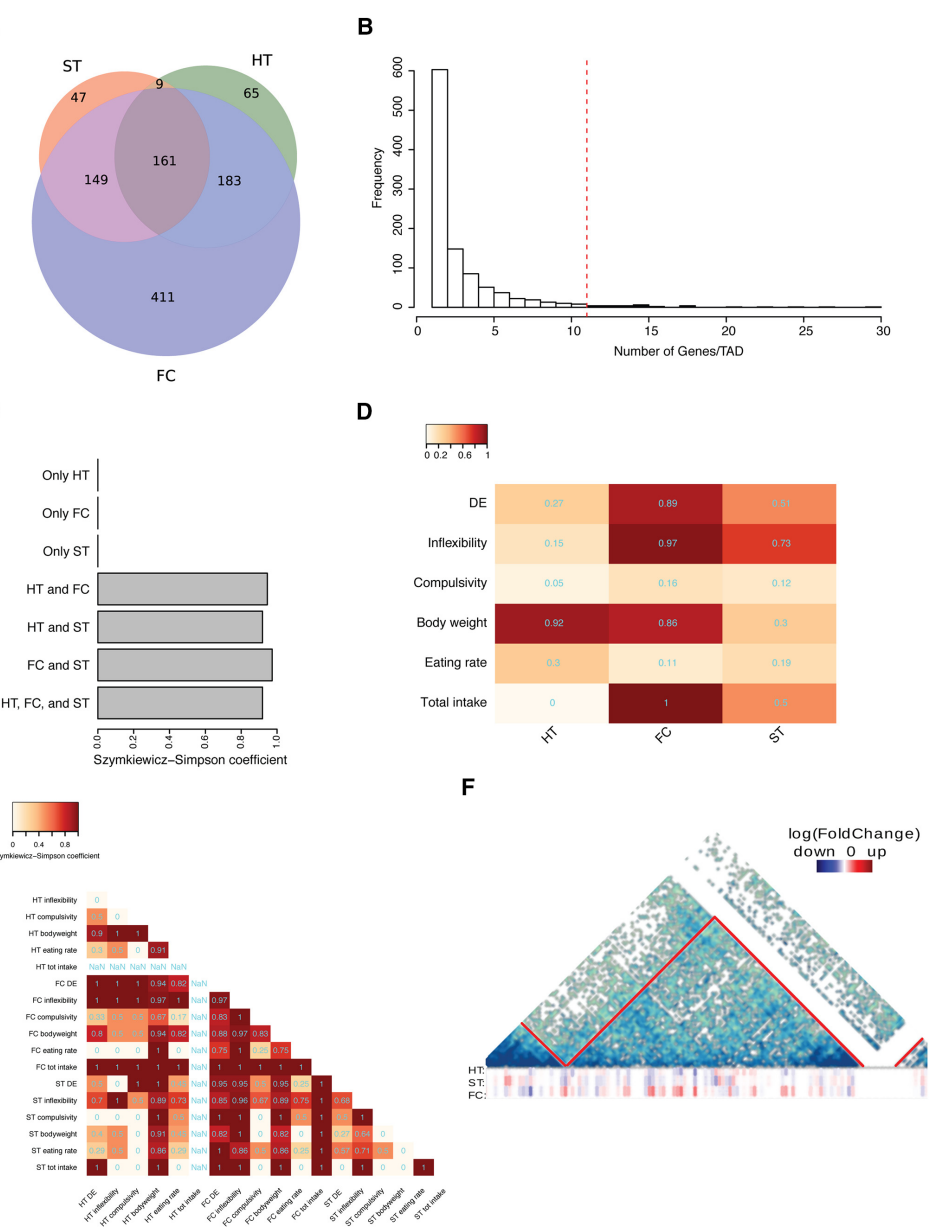
Coregulation of genes within TADs. ***A***, Venn diagram showing the overlap among TADs containing at least one DE or correlating gene in the three brain areas. Colors represent the same brain areas as in Figure 2*A*. Circles’ areas are proportional to the gene counts. ***B***, Histogram showing the number of regulated genes per regulated TADs. The dashed red line demarks the 5% area of the distribution with TADs containing high number of regulated genes. Bars corresponding to these regulated TADs are on the right of the dashed line. ***C***, Barplot showing the Szymkiewicz−Simpson overlap coefficient between regulated TADs with region-specific or coregulated TADs. Brain region acronyms are the same as in Figure 2. ***D***, Heatmap showing the Szymkiewicz−Simpson overlap coefficient between regulated TADs and TADs containing any of the DE or genes correlating with phenotypical variables (rows) in the three examined regions (columns). Brain region acronyms are the same as in Figure 2. Color code according to the coefficient. ***E***, For each group of DE or correlating genes, we considered the subset of the 37 TADs on which the respective genes were mapping. The heatmap shows the overlap among those regulated TADs for each DE gene list, phenotypical variable, and brain region. The color code is proportional to the Szymkiewicz−Simpson overlap coefficient, which is also printed in cyan on the cells. ***F***, Hi-C map of TAD 624–example of a regulated TAD. TAD 624 is located on the chromosome 7: 109600000–113000000 bp. Three heatmaps at the bottom of the TAD represent expression of the genes localized within this TAD. Red color depicts upregulation and blue color downregulation of the particular gene. HT indicates the hypothalamic region, ST the striatum region, FC the frontal cortex, and DE as differentially expressed. Extended information related to the differential gene expression and correlating genes conformed within the TADs structure could be found in Extended Data Figures 4-1, 4-2.

10.1523/ENEURO.0287-18.2018.f3-4 Extended Data Figure 3-4 Genes correlating with body weight. ***A***, Dot plot showing the ρ score for body weight (*x*-axis) and the log2FC (*y*-axis) in frontal cortex. Each dot is a microarray probe. ***B***, Barplot showing how many genes with an absolute ρ higher than 0.9 for a given bin of log2FC. ***C***, Same as in ***B*** normalized for the total number of genes present in a given bin of log2FC. ***D–F***, Same as in ***A–C*** for striatum. ***G–I***, Same as in ***A–C*** for hypothalamus Download Figure 3-4, TIF file.

**Figure 5.**
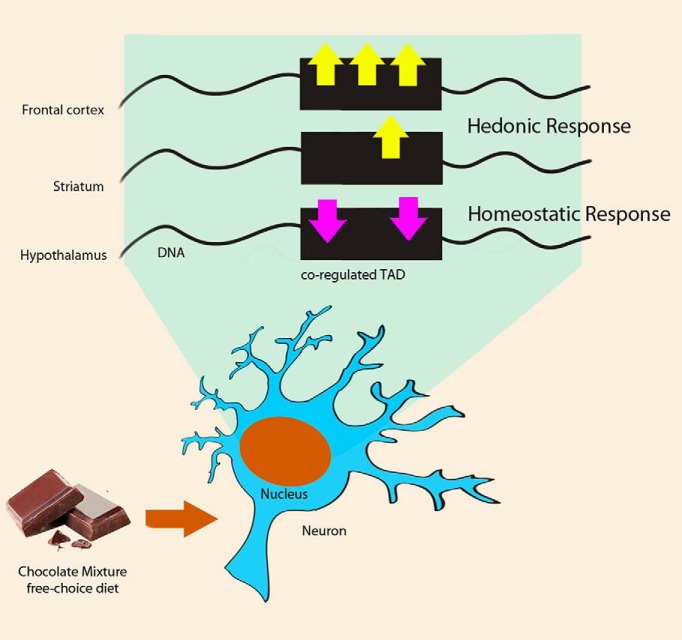
TADs orchestrate the transcriptional response both within and across brain areas. Cartoon depicting the response at the TADs level on free choice CM diet, in the nucleus of frontal cortex, striatum, or hypothalamus neurons. Differentially coregulated TADs is simplified as a black box, with yellow arrows standing for upregulated genes, and purple ones downregulated genes.

10.1523/ENEURO.0287-18.2018.f3-5 Extended Data Figure 3-5 Genes correlating with eating rate. ***A***, Dot plot showing the ρ score for eating rate (*x*-axis) and the log2FC (*y*-axis) in frontal cortex. Each dot is a microarray probe. ***B***, Barplot showing how many genes with an absolute ρ higher than 0.9 for a given bin of log2FC. ***C***, Same as in ***B*** normalized for the total number of genes present in a given bin of log2FC. ***D–F***, Same in ***A–C*** for striatum. ***G–I***, The same in ***A–C*** for hypothalamus Download Figure 3-5, TIF file.

10.1523/ENEURO.0287-18.2018.f3-6 Extended Data Figure 3-6 Genes correlating with total intake. ***A***, Dot plot showing the ρ score for total intake (*x*-axis) and the log2FC (*y*-axis) in frontal cortex. Each dot is a microarray probe. ***B***, Barplot showing how many genes with an absolute ρ higher than 0.9 for a given bin of log2FC. ***C***, Same as in ***B*** normalized for the total number of genes present in a given bin of log2FC. ***D–F***, Same as in ***A–C*** for striatum. ***G–I***, Same as in ***A–C*** for hypothalamus Download Figure 3-6, TIF file.

10.1523/ENEURO.0287-18.2018.f3-7 Extended Data Figure 3-7 DE and correlating genes are enriched in region-specific molecular pathways ***A***, REACTOME enrichment analysis for DE and correlating genes in the three brain areas. The color gradient indicates the adjusted *p* value for the enrichment. Number in parentheses indicates the number of identified genes in each category. Dot size corresponds to (gene count for each group)/(total gene count for each category). In case of overlapping categories, only the most significant one is shown (see Materials and Methods). ***B***, KEGG enrichment analysis for DE and correlating genes in the three brain areas. The color gradient indicates the adjusted *p* value for the enrichment. Numbers in parentheses indicate the number of identified genes in each category. Dot size corresponds to (gene count for each group)/(total gene count for each category). In case of overlapping categories, only the most significant one is shown (see Materials and Methods). ***C***, GO enrichment analysis for DE and correlating genes in the three brain areas. The color gradient indicates the adjusted *p* value for the enrichment. Numbers in parentheses indicate the number of identified genes in each category. Dot size corresponds to (gene count for each group)/(total gene count for each category). In case of overlapping categories, only the most significant one is shown (see Materials and Methods). Only pathways with an FDR < 5% are shown. Download Figure 3-7, TIF file.

10.1523/ENEURO.0287-18.2018.f4-1 Extended Data Figure 4-1 DE and correlating genes conformed within the TADs structure. ***A***, Kruskal–Wallis tests *H* statistics illustrating distribution of variance of gene expression fold changes among TADs (red dots) and the permuted gene expressions in the frontal cortex (FC), hypothalamus (HT), and striatum (ST). ***B***, Kruskal–Wallis tests *H* statistics illustrating distribution of variance of gene expression fold changes among TADs (red dots) and correlations of gene expressions with phenotypical variables (other colors) in the frontal cortex (FC), hypothalamus (HT), and striatum (ST). Download Figure 4-1, TIF file.

Most of the genes highly correlating with behavioral variables showed subtle expression changes [average absolute log2FC of ∼0.2–0.4; Fig. 3*B*; Extended Data Figs. 3-2, 3-3, 3-4, 3-5, 3-6]. When plotting the log2FC as a function of the Spearman’s ρ, we observed a high correlation with phenotypic variables for genes changing <1.5 times (ρ range: 0.27–0.86), and a low correlation for genes changing >1.5 times (ρ range: 0–0.25).

Instead, only few DE genes were significantly correlating with behavior or body weight, as demonstrated by the low overlaps between DE genes and correlating genes (Fig. 3*C*). The most relevant overlaps were found between hypothalamic DE genes correlating with body weight (31%) and frontal cortex DE genes correlating with inflexibility (13.7% of correlating genes). Overall, 79% of frontal cortex DE genes, 96% of striatum DE genes, and 66% of hypothalamic genes were not correlating with any of our studied variables, indicating that DE and correlating genes are two different categories of regulated genes.

Contrary to DE genes, which were shared across brain regions with quite high overlap (frontal cortex DE genes with striatal and hypothalamic DE genes), genes correlating with a given phenotypic variable were not the same across the three brain regions (Fig. 3*C*) with low overlaps both intrabrain (among phenotypical variables) and interbrain region. This suggests the need of activation of both common and region-specific transcriptional programs in each brain area, for each phenotypic change to occur. Two exceptions were the overlap of genes correlating with total intake and inflexibility in the frontal cortex (46%) and genes correlating with inflexibility and compulsivity in the striatum (63%; Fig. 3*C*).

### Transcriptional changes affect both common and region-specific molecular pathways

We then investigated the molecular pathways (Extended Data Fig. 3-7*A* for Reactome and 8B for KEGG) and gene ontologies (Extended Data Fig. 3-7*C* ) enrichment of both DE genes (changing their expression >1.5–2 times) and genes significantly correlating with phenotypic variables (mainly showing more modest log2 FCs of 0.2–0.4) in the three brain areas.

In the hypothalamus DE genes were mainly enriched in Reactome’s “olfactory signaling pathways” (Extended Data Fig. 3-7*A* ), KEGG’s “olfactory transduction” (Extended Data Fig. 3-7*B* ) and GOs “olfactory receptor activity” and “sensory perception of smell” (Extended Data Fig. 3-7*C* ). Hypothalamic genes correlating with inflexibility were mainly enriched in the Reactome “endosomal/vacuolar pathway,” and the metabolic pathway “translocation of GLUT4 to the plasma membrane” (Extended Data Fig. 3-7*A* ) categories and several GOs related to the metabolism of fatty acids and sugars such as lactonase, hydrolase, mannosidase, and esterase activity (Extended Data Fig. 3-7*C* ). Finally, hypothalamic genes correlating with body weight showed enrichments mainly in epigenetic/chromatin pathways, indicating they are tightly regulated at the transcriptional level.

Frontal cortex DE genes, similarly to hypothalamic ones, were also enriched in olfactory transduction and “taste transduction” pathways (Extended Data Fig. 3-7*B* ), together with olfactory receptor activity and “sensory perception of chemical stimulus.” Frontal cortex genes correlating with inflexibility were similarly enriched in olfactory transduction (Extended Data Fig. 3-7*B* ) and olfactory receptor activity (Extended Data Fig. 3-7*C* ), consistently with the overlap between inflexibility genes and frontal cortex DE genes (Fig. 3*C*). Finally, frontal cortex genes correlating with compulsivity were enriched in the immunity pathway “α-defensins” (Extended Data Fig. 3-7*A* ). Taken together, the results indicate that genes belonging to olfactory transduction related pathways are commonly deregulated in both the hypothalamus and the frontal cortex, where part of these genes is also highly correlating with inflexibility.

Regarding the striatum, genes correlating with inflexibility and compulsivity shared enriched categories, as expected by their high overlap of 63% (Fig. 3*C*), suggesting that compulsivity and inflexibility are connected processes in the striatum, involving pathways such as “alcoholism” (Extended Data Fig. 3-7*B* ) and chromatin pathways mainly related to gene silencing (Extended Data Fig. 3-7*C* ). Other striatal genes such as genes correlating with eating rate were enriched in “glyoxylate and dicarboxylate metabolism” (Extended Data Fig. 3-7*B* ), while genes correlating with body weight with GOs “mitochondrial membrane” (Extended Data Fig. 3-7*C* ). Finally, genes correlating with total intake in the striatum were both enriched with nuclear/transcriptional pathways and immune pathways related with leukocytes (Extended Data Fig. 3-7*A*,*C* ). Summary tables for the enrichment analysis are reported as Extended Data 3-1, 3-2, 3-3.

10.1523/ENEURO.0287-18.2018.ed3-1 Extended Data 3-1 Results for the REACTOME enrichment analysis. Download Extended Data 3-1, CSV file.

10.1523/ENEURO.0287-18.2018.ed3-2 Extended Data 3-2 Results for the KEGG enrichment analysis. Download Extended Data 3-2, CSV file.

10.1523/ENEURO.0287-18.2018.ed3-3 Extended Data 3-3 Results for the Gene Ontology enrichment analysis. Download Extended Data 3-3, CSV file.

### Gene expression changes are organized within regulatory domains

The analyses above showed that the transcriptional responses involve both commonly regulated and brain region-specific genes. Recently published results showed that genes lying within the same TADs have stronger correlation in expression than genes separated by TAD borders (Ramírez et al., 2018) and that actively expressed open chromatin regions are spatially separated from inactive ones (Rennie et al., 2018). However, it has not been shown whether changes in gene expression caused by a stimulus such as the different diets before the test battery in our experimental design would conform to the TAD structure as well or would be TAD independent. Also, it has not been shown that such changes would occur in the mammalian brain. Therefore, to verify if such regulation could take place, we tested whether the genes conformed to a common regulatory domain (TAD) structure in each of the investigated brain areas. To this purpose, we used the segmentation of mouse chromosomes into 1519 TADs as determined by Dixon et al. (2012) based on Hi-C experiments in cortical tissue (Extended Data 4-1).

10.1523/ENEURO.0287-18.2018.ed4-1 Extended Data 4-1 Mapping between the probes of the microarray and the TAD analyzed in our study. Only uniquely mapping probes were considered. Download Extended Data 4-1, CSV file.

We compared the distributions of all gene expression fold changes within TADs with the Kruskal–Wallis test. The distributions of fold changes across TADs were significantly different: in all brain areas, the *p* values of the Kruskal–Wallis test were lower than 10^−37^(Extended Data Fig. 4-1*A*). To assess the robustness of these significant *p* values we performed two different permutation tests. First, we reassigned *in silico* genes to TADs, therefore completely changing the published topological organization (Dixon et al., 2012). This led to a dramatic drop of the *H* statistics (for instance in the hypothalamus from *H* = 1991, *p* = 6.7E^−47^ to average *H* of 1000 permutations = 1157, *p* = 0.5), indicating that our results were specific for the specific TAD structure in the brain. Secondly, we applied a subtler permutation where we reshuffled randomly TAD boundaries while keeping the original gene positions. As expected, in this case *p* values were less severely affected than in the previous permutation (e.g., for hypothalamus, decrease from *H* = 1991, *p* = 6.7E^−47^ to average *H* statistics of the 1000 permutations *H* = 1665, *p* = 2.1E^−27^; Extended Data Fig. 4-1*A*).

We also investigated whether the distribution of the ρ values for each of our phenotypical variables (body weight, compulsivity, inflexibility, energy intake, and eating rate) agreed with the TADs segmentation pattern. Again, the distribution of ρ values was not random across TADs, indicating that the correlation values were not uniformly distributed but tended to cluster in agreement with the TAD structure (e.g., for hypothalamus, eating rate, *H* = 2514, *p* = 6.23E^−102^, as compared to average of the 1000 permutations: *H* = 1154, *p* = 0.5; Extended Data Fig. 4-1*B*).

Overall, these analyses suggest that both gene expression changes between SC and CM groups and correlation values between genes and phenotypic variables occur in conformity with the brain TAD structure.

### A high number of TADs were simultaneously coregulated across the three brain regions

To determine whether TADs were involved in the coordination of region-specific transcriptional responses, we analyzed all the TADs containing regulated genes, both DE and correlating, for each brain area. These TADs overlapped widely across the three brain regions, with 502 TADs shared in at least two brain regions and 161 across the three brain regions (Fig. 4*A*). Concordant to its higher and wider transcriptional response, the frontal cortex contained the highest number of region-specific TADs. However, when looking at the number of genes per TADs, most of the regulated genes were contained in the same TADs across brain areas. This held true also for the frontal cortex, in which although we found a higher number of region-specific TADs, most of the regulated genes mapped to common TADs (Extended Data Fig. 4-2*A*).

10.1523/ENEURO.0287-18.2018.f4-2 Extended Data Figure 4-2 (A). Heatmap showing the percentages of counts of the genes contained in the TADs set from Fig 4*A* over the DE gene and phenotypical variables for each brain area. Actual gene numbers are printed in cyan. (B) Left side. Heatmap where each row corresponds to a regulated TAD, each column to a brain region. The color code indicates the difference between upregulated and downregulated genes number (considering only DE and correlating genes), from yellow (more up- regulated genes), to violet (more downregulated genes), passing for white (equal number). Gray boxes are TADs without any regulated genes for that specific region. Right side. Heatmap where each column corresponds to DE and correlating genes for each brain region, and each row to a regulated TADs. The color code indicates the actual number of genes per each TADs in a given category. Download Figure 4-2, PDF file.

The number of regulated genes per TAD followed a heavy-tailed distribution with hundreds of TADs containing only one or few regulated genes and a long tail with few TADs highly enriched in regulated genes (Fig. 4*B*). This tendency was significant, as verified by permutation testing.

Considering the overall area of this distribution of TADs as 1, we named regulated TAD*s* (*n* = 37; Fig. 4*B*, bars on the right of the dashed line) those in the tail on the right of the graph (cutoff for the area of 0.05). These TADs contained >10 genes coregulated either within a specific brain region and/or among regions. Interestingly, all regulated TADs contained genes regulated in at least two different brain areas, and >90% of them contained genes regulated across all the three studied brain areas (Fig. 4*C*).

In the hypothalamus, genes correlating with body weight were mainly localized in regulated TADs suggesting that genes in these TADs are needed for body weight regulation (Fig. 4*D*). In the frontal cortex, regulated TADs showed the highest enrichments in DE genes, and in genes correlating with inflexibility, body weight, and total intake. In the striatum regulated TADs were also enriched in genes correlating with inflexibility (Fig. 4*D*).

We detected transcriptional coregulation both within and across brain regions. Within brain regions, many regulated TADs contained at the same time genes correlating with different phenotypical variables (e.g., regulated TADs containing compulsivity genes and inflexibility genes in frontal cortex). Across brain regions, regulated TADs contained genes correlating with phenotypical variables in at least two or three brain regions (e.g., inflexibility genes or body weight genes across the three brain regions; Fig. 4*E*).

Since TADs would provide the epigenetic environment for coexpression of groups of genes, upregulated and downregulated genes may cluster separately in certain regulated TADs. In fact, differences between upregulated and downregulated genes per TAD often deviated from zero (Extended Data Fig. 4-2*B*, left side). These deviations were higher in frontal cortex with a group of regulated TADs containing mainly upregulated genes and another group containing mainly downregulated genes. In the hypothalamus, almost the all regulated TADs contained downregulated genes, while the striatum showed much lower deviations in the number of upregulated and downregulated genes per TAD (Extended Data Fig. 4-2*B*, left side). These deviations were significant for the frontal cortex (mean difference per TADs between the number of upregulated and downregulated genes of 3.78, *p* = 0.02), and for the hypothalamus (mean deviation of 2, *p* = 0.002), but not for the striatum (mean deviation of 1.14, *p* = 0.7; for a detailed explanation of the permutation test used, see Materials and Methods). These results indicate that in both the frontal cortex and the hypothalamus, responsive genes distribute accordingly to their fold change along the regulated TADs, showing intra-TAD coregulation.

Interestingly, over 70% of regulated TADs contained genes upregulated in the frontal cortex, and downregulated in the hypothalamus, supporting the idea that in some cases TADs are regulated differently depending on the brain area. Each regulated TAD contained genes correlating to different phenotypical variables or DE genes (Extended Data Fig. 4-2*B*, right side), suggesting that these functions could be finely regulated in space and time thanks to the TAD organization.

One example of a TAD containing coregulated genes is TAD 624 (Fig. 4*F*), with a group of genes mainly upregulated in the frontal cortex, mainly downregulated in the hypothalamus, and with less evident intra coregulation in the striatum (clusters of blue or red bars).

## Discussion

In this work, we were interested in understanding the mechanisms of transcriptional responses comparing mice receiving two different diet regimes, SC versus energy-dense, free choice diet, in brain regions involved in the homeostatic and hedonic control of feeding behavior.

The transcriptional profile in the frontal cortex, striatum, and hypothalamus was modified consistently with the transcriptional associated domain (TAD) segmentation pattern. We detected two levels of transcriptional regulation: a switch-like regulation with DE genes changing over 1.5-fold; and a “fine-tuned” gene regulation, with subtler expression changes but highly correlated with body weight gain and behavioral changes. Although the modulation of many genes was brain region specific, mapping of the transcriptional response at the TAD level revealed many TADs that were responsive (contained DE or correlating genes) in more than one brain area. Interestingly, the 37 TADs containing the highest number of regulated genes were common across brain areas. In most cases, genes in a given TAD were upregulated in one brain area and downregulated in another, indicating the importance of the TAD structure for achieving both a coordinated and brain area-specific response.

We conclude that the conserved TAD structure from Dixon et al. (2012), participates in orchestrating gene regulation within and among brain regions controlling energy intake and reward, probably allowing a coordinated homeostatic and hedonic response.

### Different physical and behavioral parameters correlate with transcription, suggesting a coordinated and specific response across brain areas

Our free-choice paradigm promoted body weight gain and meal pattern and behavioral changes in mice. In our microarray experiment, the hypothalamus showed a remarkably different transcriptional response compared to the striatum and the frontal cortex as revealed by multidimensional scaling. This would support the different role of the hypothalamus, which controls the homeostatic regulation, from the frontal cortex and the striatum, which control the hedonic regulation of appetite. This first approach used classical differential gene expression analyses that only consider those gene expression changes satisfying specific criteria of fold change and within-group variance (Phipson et al., 2016). However, thanks to our experimental design, we could directly test the correlation of gene expression with body weight and behavioral measurements. In fact, since we collected the brain samples 6 d after the test battery, our observed gene expression profiles might not only be the result of the diet (SC or CM) but also of the interaction of the chronic effect of the diet regime with the battery test performed (for example, a gene could be DE when comparing the CM and the SC groups but only after the two groups undergo the test battery). Of course, our experimental design does not allow to disentangle the respective contribution on gene expression of the diet, the behavioral battery, or their interaction, but that goes beyond our aim. What we can state is that whether an interaction between the effects of the diet and the behavioral battery occur or not, in both cases the observed differences would be triggered by the different diet regimes, since the test battery is performed in the exact same way for the two groups, and therefore would cancel out when computing the CM-SC contrast. This original approach revealed genes highly correlated with the phenotype, that otherwise would have been filtered out for having too subtle absolute differential expression fold changes and/or too high intra-group variability. To reduce the biases related to single variables, in the case of variables characterised by multiple types of measurements, we correlated the first principal component instead of single variables. For example, compulsivity is a complex behavioral domain that is reflected in increased grooming, impaired nesting behavior, increased overeating (energy intake) across days, especially when access to energy-dense diet is restricted, and inflexible behavior in the quinine adulteration test. We speculate that genes correlating directly to a given phenotypical variable are responding to our experimental design even if in a subtler way. Remarkably, the number of correlating genes varied significantly among brain areas in accordance with their distinct biological role in feeding behavior regulation. For example, inflexibility correlated with hundreds of genes in the frontal cortex and the striatum, the brain areas that are mainly responsible for this behavior, but not in hypothalamus, whose genes mainly correlated with body weight. This fits with the hypothalamic role in the homeostatic control of energy intake (Sisley and Sandoval, 2011).

Given the importance of this finding, we included both DE and correlating genes in our pathway analysis. Among the most significant pathways we found GO enrichment in “olfactory signaling-related processes” when analyzing DE genes in the frontal cortex and in the hypothalamus, and genes correlating with inflexibility in frontal cortex. There are >1000 olfactory receptor genes in the mouse genome, that encode G-protein-coupled receptor that work as chemical sensors in the brain (Garcia-Esparcia et al., 2013). Interestingly, among the natural ligands of those olfactory receptors are fatty acid derivatives (Sartorius et al., 2015) that would be increased by our CM diet. Other categories found consistently enriched are related to the immune response. For instance, compulsivity genes in frontal cortex were enriched in defensins and total intake genes in the striatum in leukocyte-related pathways. In line with this, it is known that obesogenic food can also induce neuroinflammation (Beilharz et al., 2015).

Moreover, according to the role of the striatum in reward and addiction, we found enrichment in the alcoholism pathway for striatal genes correlating with inflexibility and compulsivity (Volkow et al., 2013). The high overlap between striatal genes correlating with inflexibility and compulsivity suggests that the processes leading to compulsive and inflexible behaviors are similar in the striatum. In this region, genes correlating with eating rate were enriched in glyoxylate and dicarboxylate metabolism, and genes correlating with body weight with mitochondrial membrane. In the hypothalamus, we found genes involved with inflexibility that were enriched in pathways involved in the metabolism of glucose and fatty acids. For example, we detected an enrichment for the translocation of the glucose transporter GLUT4 on the plasma membrane, a pathway normally activated by insulin to allow the uptake of glucose from the bloodstream (Muretta and Mastick, 2009).

Finally, many categories involved in chromatin, epigenetic, and transcriptional regulation were specifically enriched when looking at genes correlating with body weight in the hypothalamus and compulsivity and inflexibility in the striatum. This suggests that these processes might be epigenetically regulated in these brain areas.

### TADs orchestrate the brain-area-specific response

In our dataset, some groups of genes, such as genes DE in frontal cortex and hypothalamus, genes correlating with inflexibility in frontal cortex, and genes correlating with compulsivity and inflexibility in the striatum were highly overlapping and shared biological pathways such as olfactory transduction. However, we also detected many region-specific genes, leading to region-specific pathway enrichments. We wondered how region-specific mechanisms would coexist with the need to coordinate different responses both intrabrain and interbrain areas.

Therefore, we explored the distribution of regulated genes along the chromosomes to identify potential regulatory mechanisms leading to the observed expression profiles. We found that both DE genes and subtly regulated genes correlating with phenotypic and behavioral variables were not randomly distributed throughout the genome but organized in genomic clusters, the TADs. The non-random organization of genes along eukaryotic chromosomes is well established and plays a role in the coordination of gene expression and, thus, might have a functional role at the transcriptional stage.

To detect the most relevant genomic regions responsive to our experimental design in brain, we focused our analysis on the TADs with the highest number of DE genes or genes correlating with some specific behavioral variables across brain areas (what we named regulated TADs). All regulated TADs contained genes responsive across brain areas, and correlating with different phenotypical variables, indicating that they are important for the regional coregulation in the brain, and for the coordination of the different responses initiated in our two groups of mice. Consistently with the homeostatic role of the hypothalamus, we found that the majority of the regulated TADs containing hypothalamic genes, contained genes correlating with body weight. Similarly, the frontal cortex or striatum genes contained in regulated TADs were correlated with inflexibility, in agreement with the role of these brain regions in the hedonic responses to food.

We observed that the DE or correlating genes contained in regulated TADs tended to have expression changes of the same sign, supporting the idea that TADs provide the epigenetic environment for coexpression of groups of genes (Tanay and Cavalli, 2013). Interestingly, many regulated TADs show a different direction of regulation depending on the brain area (the same TAD could contain for example genes mainly upregulated in one brain area and mainly downregulated in another).

The fact that the same TADs contain genes coregulated within a brain area and regulated in different directions across brain areas might be surprising at first but is consistent with the “epigenetic plasticity” model, for which a permissive or “plastic” chromatin state activate regulatory programs (Flavahan et al., 2017). Based on our findings, we could speculate that these regulated TADs are thus the genome regions of highest epigenetic plasticity.

### Conclusions, limitations, and future direction

Our results support the hypothesis that the homeostatic and hedonic control of eating behavior could be coordinated thanks to TADs inducing a specific and coordinated transcriptional changes both intrabrain and interbrain areas (Fig. 5). Of course, we cannot discard that the test battery itself affects the transcriptional profile; nonetheless the changes should affect similarly the CM group and the SC group.

Also, we cannot rule out the possibility that our experimental design could affect the TAD structure, but given the fact that the domain structure is mainly stable (Barutcu et al., 2015), we assumed that the TAD boundaries remained intact. Expectedly, permuting those borders just slightly increased the *p* values associated with the Kruskal–Wallis test (but statistical significance was preserved). Finally, brain regions contain different cell types and we observe only the “final” averaged effect. Single-cell RNA sequencing or separation tagged cell populations could be used to assess which are the main cell subtype which are responding to the energy-dense diet. Our findings warrant future studies directly aimed to detect changes in the 3D genome organization on energy-dense diet.
