# Supplementary figures and images for: Overweight Mice Show Coordinated Homeostatic and Hedonic Transcriptional Response across Brain
Source: eNeuro. 2019 Jan 8;5(6):ENEURO.0287-18.2018. doi: 10.1523/ENEURO.0287-18.2018 (PMC6327943; doi:10.1523/ENEURO.0287-18.2018)

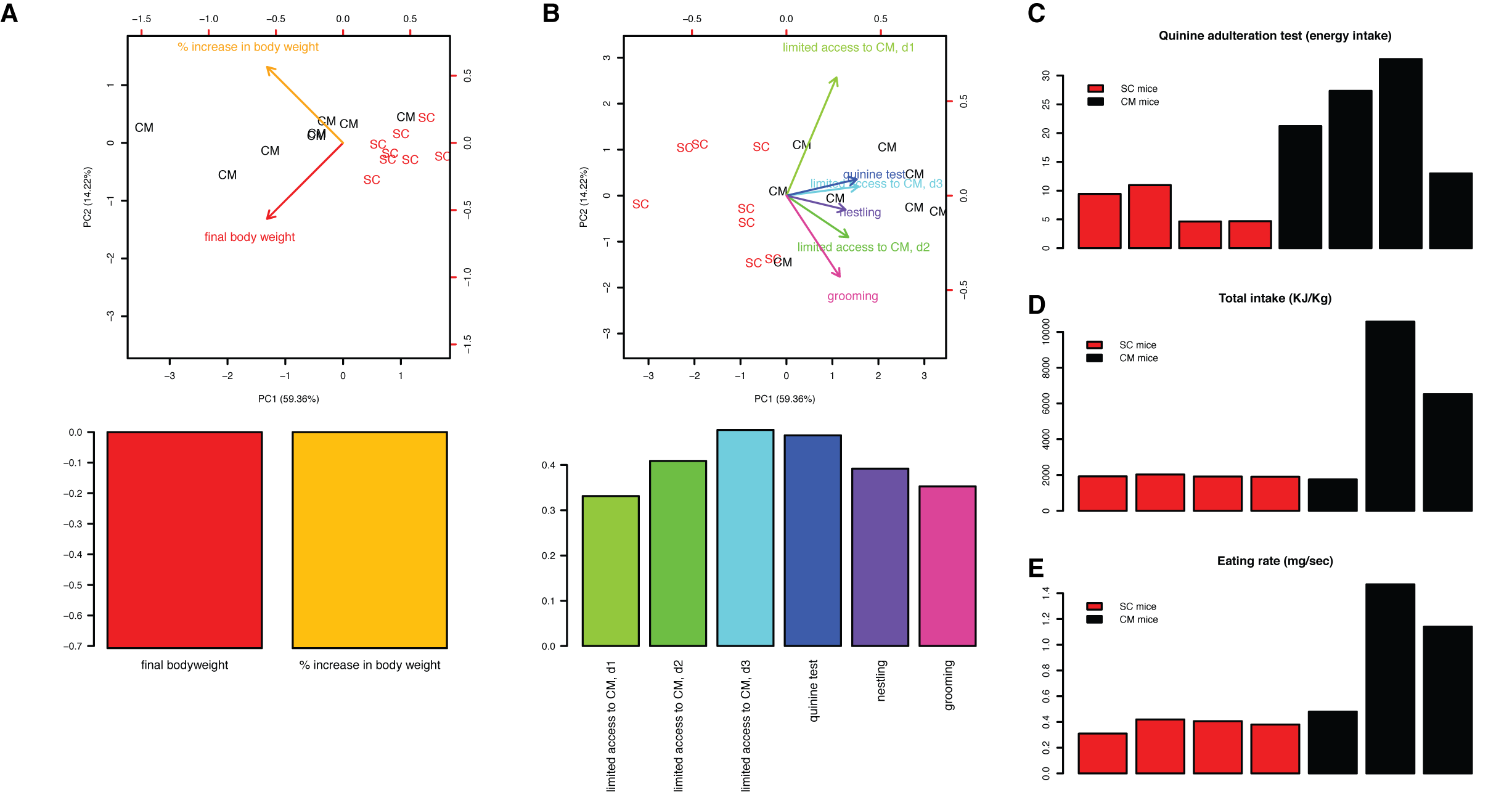

Supplement: Extended Data Figure 3-1 — Correlation of transcriptional with behavioral analysis. A, Biplot of PCA on SC and CM mice using body weight variables indicated by colored arrows (top) showing the clear separation between CM and SC mice. Barplot showing the contribution of the body weight variables to the principal component 1 (bottom). B, Biplot of PCA on SC and CM mice using behavioral variables indicated by colored arrows (top). Barplot showing the contribution of behavioral variables to the principal component 1 (bottom). C–E, Individual values of the variables used for gene expression correlation in SC mice (red) and CM mice (black). Note that eating rate and total intake values were only available for three of the four individuals used for the transcriptome analysis. C, Barplot showing the differences in CM energy intake of SC and CM mice in the quinine adulteration test. D, Barplot showing the differences in total energy intake of SC and CM (CM mice). Data from one of the mice in the CM group were missing. E, Barplot showing the differences in eating rate of SC (SC mice) and CM (CM mice). Download Figure 3-1, TIF file. [file sup_enu-eN-NWR-0287-18-s01.tif]

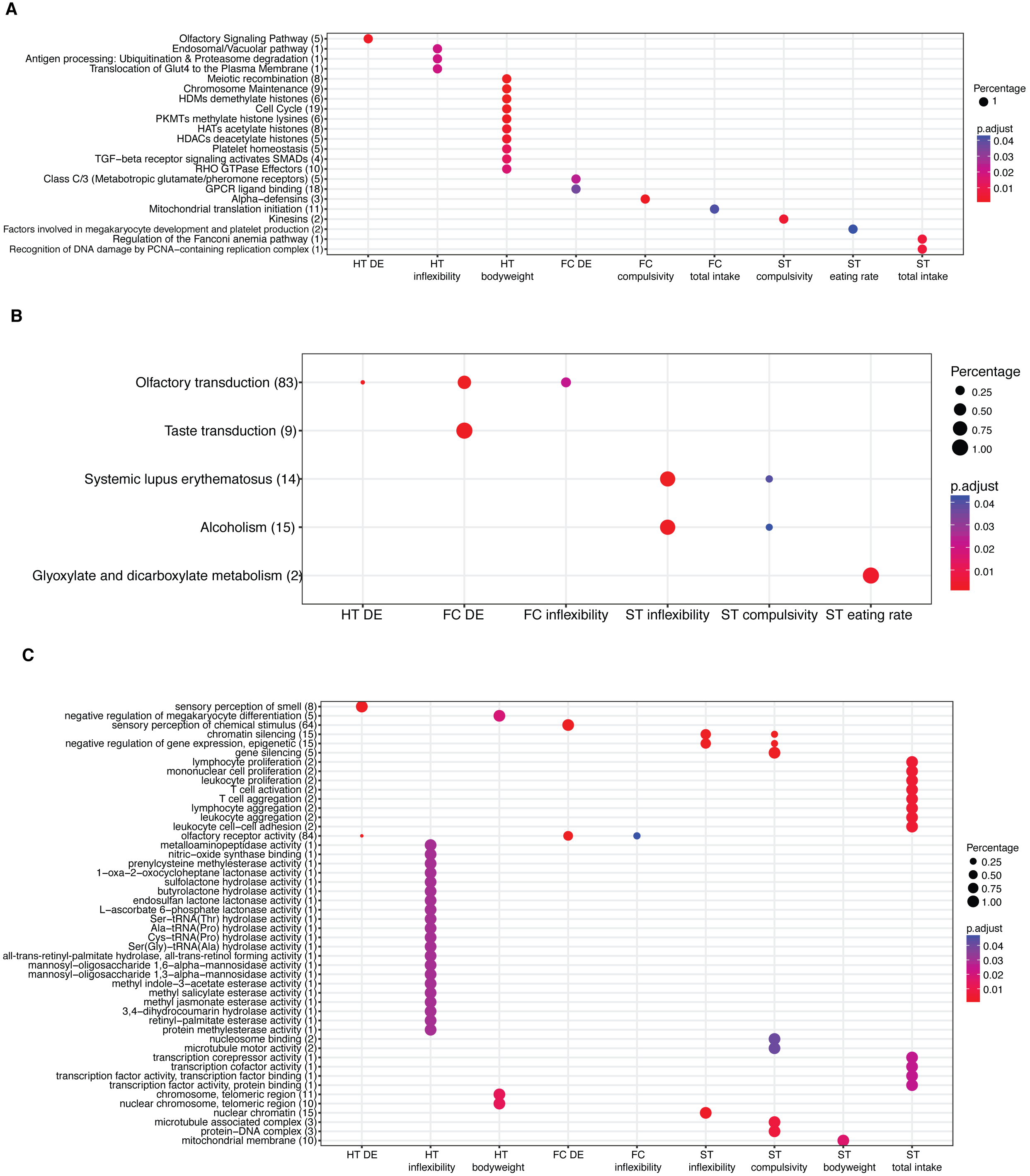

Supplement: Extended Data Figure 3-7 — DE and correlating genes are enriched in region-specific molecular pathways A, REACTOME enrichment analysis for DE and correlating genes in the three brain areas. The color gradient indicates the adjusted p value for the enrichment. Number in parentheses indicates the number of identified genes in each category. Dot size corresponds to (gene count for each group)/(total gene count for each category). In case of overlapping categories, only the most significant one is shown (see Materials and Methods). B, KEGG enrichment analysis for DE and correlating genes in the three brain areas. The color gradient indicates the adjusted p value for the enrichment. Numbers in parentheses indicate the number of identified genes in each category. Dot size corresponds to (gene count for each group)/(total gene count for each category). In case of overlapping categories, only the most significant one is shown (see Materials and Methods). C, GO enrichment analysis for DE and correlating genes in the three brain areas. The color gradient indicates the adjusted p value for the enrichment. Numbers in parentheses indicate the number of identified genes in each category. Dot size corresponds to (gene count for each group)/(total gene count for each category). In case of overlapping categories, only the most significant one is shown (see Materials and Methods). Only pathways with an FDR < 5% are shown. Download Figure 3-7, TIF file. [file sup_enu-eN-NWR-0287-18-s07.tif]

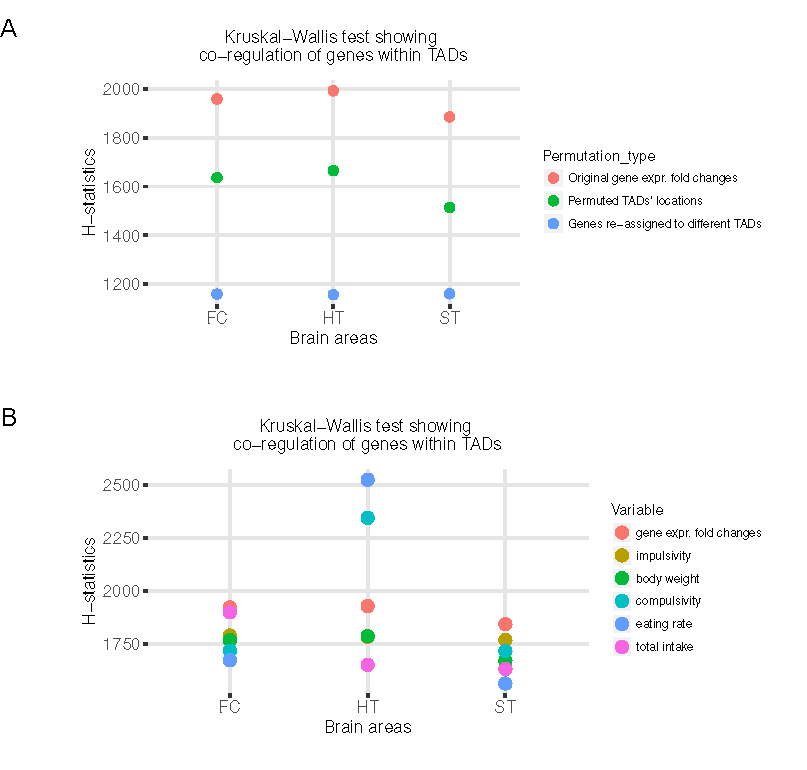

Supplement: Extended Data Figure 4-1 — DE and correlating genes conformed within the TADs structure. A, Kruskal–Wallis tests H statistics illustrating distribution of variance of gene expression fold changes among TADs (red dots) and the permuted gene expressions in the frontal cortex (FC), hypothalamus (HT), and striatum (ST). B, Kruskal–Wallis tests H statistics illustrating distribution of variance of gene expression fold changes among TADs (red dots) and correlations of gene expressions with phenotypical variables (other colors) in the frontal cortex (FC), hypothalamus (HT), and striatum (ST). Download Figure 4-1, TIF file. [file sup_enu-eN-NWR-0287-18-s08.tif]
