# Supplementary material for: Overweight Mice Show Coordinated Homeostatic and Hedonic Transcriptional Response across Brain
Source: eNeuro. 2019 Jan 8;5(6):ENEURO.0287-18.2018. doi: 10.1523/ENEURO.0287-18.2018 (PMC6327943; doi:10.1523/ENEURO.0287-18.2018)
Supplement: Extended Data Figure 4-2 — (A). Heatmap showing the percentages of counts of the genes contained in the TADs set from Fig 4A over the DE gene and phenotypical variables for each brain area. Actual gene numbers are printed in cyan. (B) Left side. Heatmap where each row corresponds to a regulated TAD, each column to a brain region. The color code indicates the difference between upregulated and downregulated genes number (considering only DE and correlating genes), from yellow (more up- regulated genes), to violet (more downregulated genes), passing for white (equal number). Gray boxes are TADs without any regulated genes for that specific region. Right side. Heatmap where each column corresponds to DE and correlating genes for each brain region, and each row to a regulated TADs. The color code indicates the actual number of genes per each TADs in a given category. Download Figure 4-2, PDF file. [file sup_enu-eN-NWR-0287-18-s17.pdf]

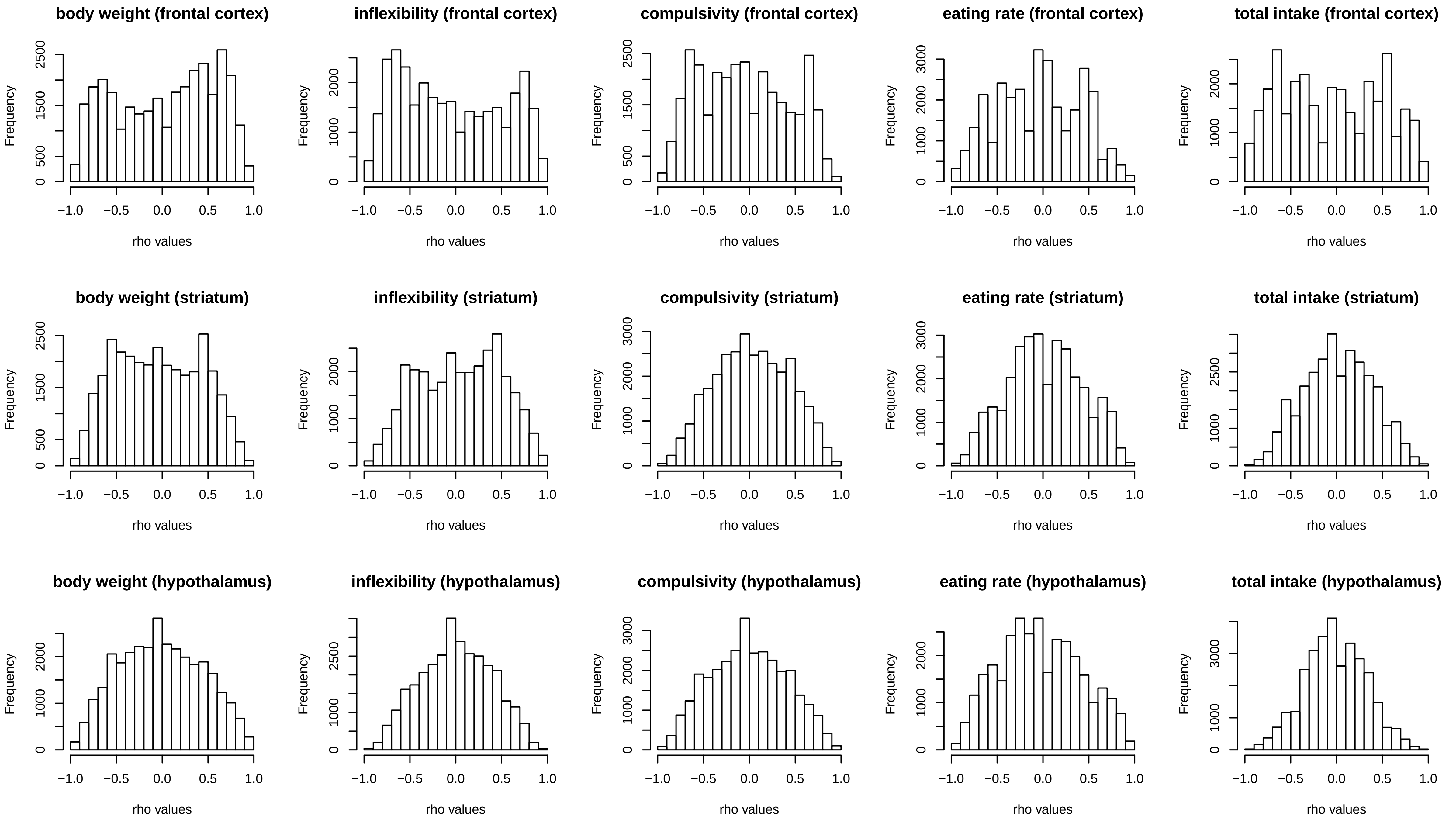

body weight (frontal cortex)
inflexibility (frontal cortex)
compulsivity (frontal cortex)
eating rate (frontal cortex)
total intake (frontal cortex)
body weight (striatum)
inflexibility (striatum)
compulsivity (striatum)
eating rate (striatum)
total intake (striatum)
body weight (hypothalamus)
inflexibility (hypothalamus)
compulsivity (hypothalamus)
eating rate (hypothalamus)
total intake (hypothalamus)
Frequency
rho values
−1.0 −0.5 0.0 0.5 1.0
